# HSP27 Interacts with Nonstructural Proteins of Porcine Reproductive and Respiratory Syndrome Virus and Promotes Viral Replication

**DOI:** 10.3390/pathogens12010091

**Published:** 2023-01-05

**Authors:** Chunhui Song, Hanze Liu, Zhi Cao, Hu Shan, Qiaoya Zhang

**Affiliations:** 1 College of Veterinary Medicine, Qingdao Agricultural University, Qingdao 266109, China; 2 China Animal Health and Epidemiology Center, Qingdao 266032, China

**Keywords:** porcine reproductive and respiratory syndrome virus (PRRSV), heat shock protein 27 (HSP27), nonstructural proteins, viral replication

## Abstract

Heat shock protein 27 (HSP27) is a multifunctional protein and belongs to the small HSP family. It has been shown that HSP27 is involved in viral replication as a cellular chaperone, but the function of HSP27 during porcine reproductive and respiratory syndrome virus (PRRSV) infections remains unexplored. Here, we found that PRRSV replication can induce HSP27 expression and phosphorylation in vitro. HSP27 overexpression promoted PRRSV replication, whereas its knockdown reduced PRRSV proliferation. Additionally, suppressing HSP27 phosphorylation reduced PRRSV replication and the level of viral double-stranded RNA (dsRNA), a marker of the viral replication and transcription complexes (RTCs). Furthermore, HSP27 can interact with multiple viral nonstructural proteins (nsps), including nsp1α, nsp1β, nsp5, nsp9, nsp11 and nsp12. Suppressing the phosphorylation of HSP27 almost completely disrupted its interaction with nsp1β and nsp12. Altogether, our study revealed that HSP27 plays an important role in PRRSV replication.

## 1. Introduction

Porcine reproductive and respiratory syndrome virus (PRRSV) is an important pathogen in pigs that can cause porcine reproductive and respiratory syndrome (PRRS). PRRSV has adversely impacted the global swine industry for more than thirty years [1]. The genome of PRRSV is a single positive-stranded RNA, approximately 15 kb in length, consisting of at least 11 open reading frames (ORFs) [2,3]. ORF1a and ORF1b, which locate at the 5’-end of the genome, encode at least 14 nonstructural proteins (nsp1α, nsp1β, nsp2, nsp3, nsp4, nsp5, nsp6, nsp7α, nsp7β, nsp8, nsp9, nsp10, nsp11, nsp12) [4,5,6]. The ORF TF and −1/−2 programmed ribosomal frameshift signal in ORF1a express nsp2N and nsp2TF [2,7]. The 3’-end of the genome includes eight small genes, which encode four non-glycosylated proteins (E, ORF5a, M, and N) and four glycoproteins (GP2a, GP3, GP4, and GP5) [8,9]. As a positive-stranded RNA virus, the viral RNA synthesis depends on the assembly of the replication and transcription complexes (RTCs), which contain both host and viral proteins as well as endoplasmic reticulum membranes [10]. The viral double-stranded RNA (dsRNA) generated during viral replication is also contained in the RTCs [11].

During PRRSV infection, protein expression in host cells can be strongly modulated. Powerful methodologies have been used to study cellular protein profiles that are modified by PRRSV. Information on the changes in proteins is crucial for identifying potential cellular factors involved in PRRSV infection. It has been observed that heat shock proteins (HSPs), including HSP70, HSP90 and HSP27, change greatly in cells following PRRSV challenge [11,12,13]. HSPs function as intracellular chaperones and are important for protein folding and assembly, proteasomal degradation, protein translocation and cell apoptosis [14]. Previous studies have demonstrated the positive effect of HSP70 and HSP90 on the replication of PRRSV [11,13]. Thus, HSP27 might also play a role in the process of PRRSV infection. At present, the role of HSP27 during PRRSV infection is unclear. 

HSP27, belonging to the small HSP family, is a multifunctional protein that participates in proinflammatory processes, cytoskeletal stability, and apoptosis inhibition [15]. HSP27 has also been reported to be involved in viral replication [16,17]. Here, we demonstrate that HSP27 and its phosphorylation were upregulated in response to PRRSV infection. Through upregulating HSP27 by transfection and downregulating HSP27 via RNA interference, it was determined that HSP27 plays an important role in PRRSV replication in cells. Suppressing HSP27 phosphorylation significantly reduced virus replication and dsRNA levels. Further analysis showed that HSP27 could interact with multiple PRRSV-nsps and that these interactions could be influenced by a specific inhibitor of HSP27 phosphorylation.

## 2. Materials and Methods

### 2.1. Cells and Viruses 

MARC-145 and HEK-293T cells were incubated in Dulbecco’s modified Eagle’s medium (DMEM) (HyClone, Logan, UT, USA) supplemented with 10% fetal bovine serum (FBS), penicillin (100 U/mL), and streptomycin sulfate (100 μg/mL). Primary porcine alveolar macrophages (PAMs) were obtained from PRRSV-negative piglets as previously described [12]. The obtained cells were cultured in RPMI 1640 medium (HyClone, Logan, UT, USA) with the same supplements as mentioned above. All the cells were cultured at 37 °C with 5% CO_2_.

The highly pathogenic PRRSV (HP-PRRSV) strain BB0907 (HQ315835.1), the classical PRRSV (C-PRRSV) strain S1 (DQ459471.1), and the NADC30-like strain FJ1402 (KX169191.1) are PRRSV-2 strains and were all kindly provided by Prof. Ping Jiang at Nanjing Agricultural University, China. The viral titer was determined by 50% tissue culture infective dose (TCID_50_) assays [18]. Ultraviolet (UV)-inactivated PRRSV was generated by exposing the BB0907 strain to shortwave UV light (254 nm) for 2 h. Heat-inactivated PRRSV was generated by incubating the BB0907 strain at 65 °C for 1 h in a water bath. Loss of infectivity was determined by detecting the virus titers.

### 2.2. Plasmid Construction

To construct the HSP27 expression vectors, genes that encode wild-type HSP27 were amplified from cDNAs of PAMs and cloned into the pcDNA3.1+ vectors. The protein was tagged with an epitope tag (HA or Flag) at the C-terminus. Expression vectors containing genes encoding PRRSV-nsps were cloned from the BB0907 strain and engineered into the pCAGGS vector. The proteins were tagged with Flag at the C-terminus.

### 2.3. Reagents and Antibodies

KRIBB3 (Abcam, Cambridge, UK) was dissolved in DMSO to 25 mM, and then stored at −80 °C. Mouse monoclonal antibodies (mAbs) against Flag or HA were purchased from Abmart (Shanghai, China). β-actin, horseradish peroxidase (HRP)-conjugated goat anti-mouse and HRP-conjugated goat anti-rabbit antibodies were purchased from Beyotime Biotechnology (Shanghai, China). Rabbit polyclonal antibodies against phospho-HSP27 (Ser15), phospho-HSP27 (Ser78), and phospho-HSP27 (Ser82) were purchased from Affinity (Changzhou, China). mAb against HSP27 was purchased from Santa Cruz Biotechnology (Santa Cruz, CA, USA). The mAb specific for dsRNA (J2) was purchased from Scicons (Sizraku, Hungary). The mAb against PRRSV-N protein was kindly provided by Prof. Ping Jiang at Nanjing Agricultural University, China.

### 2.4. RNA Extraction and Quantitative Real-Time PCR (qRT–PCR)

The total RNA of cultured cells was extracted using a Total RNA extraction Kit (Bioer Technology, Hangzhou, China). RNA was then reverse transcribed into cDNA using a HiScript II 1st Strand cDNA Synthesis Kit (Vazyme, Nanjing, China). qRT–PCR assay was carried out using AceQ^®^ qPCR SYBR^®^ Green Master Mix (Vazyme, Nanjing, China) in a LightCycler96 System. The data are presented as the fold change in gene expression normalized to β-actin and are relative to the control group. All experiments were performed three times independently. 

### 2.5. Cytotoxicity Assay

Cells in 96-well plates were treated with different concentrations of KRIBB3 or DMSO. Considering the half-life of KRIBB3, the medium was refreshed every 24 h. After 48 h of incubation, cell viability was analyzed using a Cell Counting Kit-8 (CCK-8) (Beyotime Biotechnology, Shanghai, China).

### 2.6. Indirect Immunofluorescence Assay

Cells were washed with PBS and fixed for 20 min with 4% paraformaldehyde at room temperature. After washing three times with PBS, the cells were permeabilized for 30 min with Immunostaining Permeabilization Solution with Saponin (Beyotime Biotechnology, Shanghai, China). The cells were then incubated with a monoclonal antibody against PRRSV-N protein (1:100) or dsRNA (1:200) overnight at 4°C. After being washed with PBS, the cells were incubated with Alexa Fluor 488-conjugated goat anti-mouse IgG (1:300) (Proteintech, Chicago, IL, USA) for 1 h at 37 °C. After an additional wash with PBS, fluorescence images were observed using fluorescence microscopy (Zeiss Vert. A1; Carl Zeiss Microscopy GmbH, Oberkohen, BW, Germany).

### 2.7. Western Blotting

Cells were washed with cold PBS and harvested using lysis buffer (Beyotime Biotechnology, Shanghai, China) containing 1mM phenylmethanesulfonyl fluoride (PMSF). The lysed samples were separated by 12.5% SDS-PAGE gels (Shanghai Epizyme Biomedical Technology, Shanghai, China), then electro-blotted onto PVDF membranes (Millipore, Boston, MA, USA). The membranes were blocked for 40 min in Protein Free Rapid Blocking Buffer (1×) (Shanghai Epizyme Biomedical Technology, Shanghai, China) and then incubated overnight with primary antibodies at 4 °C. After being washed with TBST (1×) (Beijing Solarbio Science & Technology, Beijing, China), the membranes were incubated with HRP-conjugated anti-mouse or anti-rabbit secondary antibodies for 45 min at room temperature. Protein bands were visualized using enhanced chemiluminescence (ECL) detection reagents (Tanon, Shanghai, China). 

### 2.8. RNA Interference

Small interfering RNAs (siRNAs) targeting HSP27 protein and a negative-control siRNA (siNC) were designed and synthesized (Sangon Biotech, Shanghai, China). The siRNA sequences were as follows: siRNA1, 5’ – AGG AUG GCG UGG UGG AGA UTT – 3’; siRNA2, 5’ – CCA CAC AGU CCA ACG AGA UTT – 3’; siRNA3, 5’ – CGA GAC UGC CGC CAA GUA ATT – 3’. The siNC sequence was 5’ -UUC UCC GAA CGU GUC ACG UTT- 3’. MARC-145 cells in 24-well plates were transfected with 50 pmol of siRNA or siNC using Lipofectamine 3000 DNA transfection reagent (Invitrogen, Carlsbad, CA, USA). To determine the efficiency of the knockdown, cells were lysed at 36 h post-transfection, and lysates were subjected to Western blot analysis.

### 2.9. Coimmunoprecipitation (Co-IP)

HEK-293T cells were grown on 6-well plates and cotransfected with plasmids that expressed HA-tagged HSP27 and Flag-tagged PRRSV-nsps using Lipofectamine 3000. At 48 h posttransfection, the cells were washed with cold PBS and lysed for 30 min on ice using NP-40 lysis Buffer (Beyotime Biotechnology, Shanghai, China) containing 1 mM PMSF. The cell lysates were centrifuged at 12,000× *g* for 10 min. For immunoprecipitation, the supernatants were incubated with mouse anti-HA mAb (Abmart, Shanghai, China) or mouse anti-Flag mAb (Abmart, Shanghai, China) at 4 °C for 4 h, followed by precipitation with Protein A+G Agarose (Beyotime Biotechnology, Shanghai, China) overnight at 4 °C with rotation. The agarose beads were harvested by centrifugation, gently washed three times with NP−40 buffer, boiled in 5× loading buffer for 5 min, and used for Western blot analysis.

### 2.10. Statistical Analysis

Data analyses were performed using GraphPad Prism 8.0 software (San Diego, CA, USA). All experiments were repeated at least three times. The results are presented as the mean ± standard deviation (SD). Statistical significance was determined by one-way analysis of variance (ANOVA) or Student’s *t*-test. *p* < 0.05 (*), *p* < 0.01 (**), and *p* < 0.001 (***) were considered statistically significant.

## 3. Results

### 3.1. PRRSV Infection Induces the Expression of HSP27

**Figure 1:**
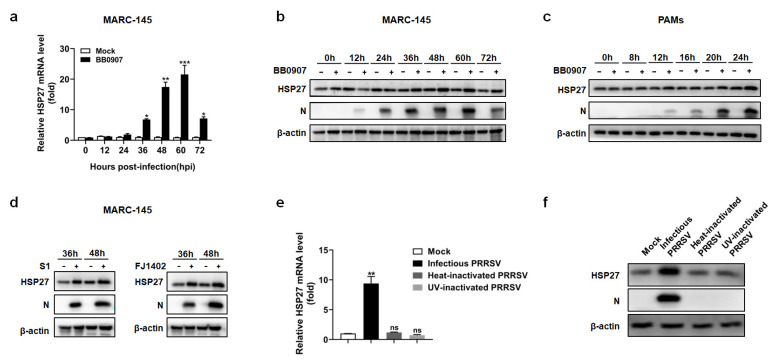
HSP27 is upregulated during PRRSV infection. (**a**–**c**) MARC-145 cells or PAMs were infected with the HP-PRRSV strain BB0907 (MOI = 1.0). Cells were harvested at various time points. HSP27 expression was analyzed by qRT–PCR (**a**) or Western blot analysis (**b**,**c**). (**d**) MARC-145 cells were infected with the C-PRRSV strain S1 or the NADC30-like strain FJ1402 (MOI = 1.0). The expression of HSP27 at different time points was analyzed by Western blot analysis. (**e**,**f**) MARC-145 cells were infected with infectious PRRSV, heat-inactivated PRRSV or UV-inactivated PRRSV (MOI = 5) for 36 h. The expression of HSP27 was determined by qRT–PCR (**e**) or Western blot analysis (**f**). *p* < 0.05 (*), *p* < 0.01 (**), and *p* < 0.001 (***) were considered statistically significant.

To evaluate the endogenous cellular HSP27 expression during PRRSV infection, MARC-145 cells were either infected or mock-infected with PRRSV BB0907. The cell lysates were collected at different hours post-infection (hpi) and then subjected to qRT–PCR and Western blot analysis, respectively. As shown in Figure 1a, the mRNA level of HSP27 was upregulated from 36 hpi. Western blot analysis revealed that HSP27 was induced from 36 hpi and viral N protein could be detected from 12 hpi (Figure 1b). We then confirmed this phenomenon in PAMs. As shown in Figure 1c, HSP27 was upregulated from 12 hpi when the N protein could also be detected.

The effects of the other two representative PRRSV strains, S1 (a classical PRRSV-2 strain) and FJ1402 (an NADC30-like PRRSV-2 strain), were also tested in MARC-145 cells. As shown in Figure 1d, both the S1 and FJ1402 strains induced HSP27 expression, which suggests that HSP27 upregulation was not dependent on PRRSV strains. These results indicated that PRRSV infection significantly upregulated the endogenous HSP27 expression in both MARC-145 cells and PAMs.

To investigate whether viral replication was necessary for the upregulation of HSP27, heat-inactivation and UV-inactivation of PRRSV were performed. Unlike infectious PRRSV, neither heat-inactivated PRRSV nor UV-inactivated PRRSV could increase HSP27 expression (Figure 1e,f). This result suggested that PRRSV replication was essential for the upregulation of HSP27.

### 3.2. HSP27 Promotes PRRSV Replication

**Figure 2:**
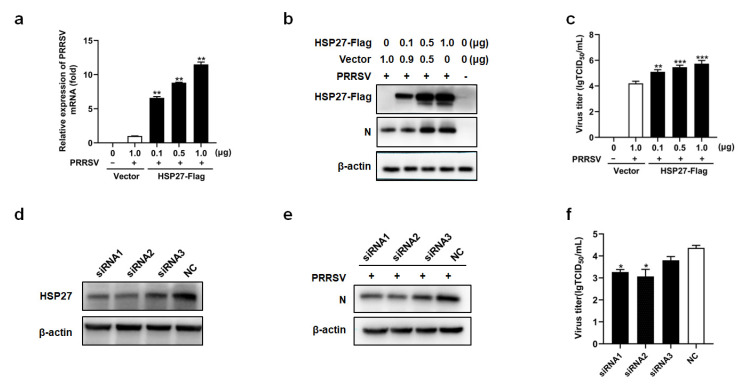
HSP27 promotes PRRSV replication. (**a**–**c**) MARC-145 cells were transfected with pcDNA3.1-HSP27-Flag or pcDNA3.1+. At 24 h after transfection, the cells were infected with PRRSV (MOI = 1.0), and after another 24 h, the cells were harvested for qRT–PCR (**a**), Western blotting (**b**) and TCID_50_ assay (**c**). (**d**) MARC-145 cells were transfected with HSP27-specific siRNAs or siNC. After 36 h, the cells were harvested to evaluate the interference efficiency by Western blotting. (**e**,**f**) MARC-145 cells were transfected with HSP27-specific siRNAs or siNC. After 36 h, the cells were infected with PRRSV (MOI = 0.1). The cells were harvested at 24 hpi to analyze PRRSV-N expression by Western blotting(e), and virus titers by TCID_50_ assays (f). *p* < 0.05 (*), *p* < 0.01 (**), and *p* < 0.001 (***) were considered statistically significant.

To explore the effects of HSP27 on PRRSV replication, overexpression and knockdown by specific siRNAs were performed with MARC-145 cells. Different doses of recombinant plasmid pcDNA3.1-HSP27-Flag or pcDNA3.1+ were transiently transfected into the MARC-145 cells. The cells were then infected with PRRSV. The results showed that PRRSV mRNA and N protein levels increased significantly with increasing pcDNA3.1-HSP27-Flag transfection doses (Figure 2a,b). Virus titers were measured by TCID_50_ assays. Consistent with the above results, PRRSV titers were also significantly increased in the pcDNA3.1-HSP27-Flag transfected groups (Figure 2c). The results indicated a direct positive correlation between HSP27 expression levels and PRRSV replication.

To further confirm the effect of HSP27 on PRRSV replication, three HSP27-targeting siRNAs were designed. The interference effects of these siRNAs were evaluated by Western blotting. As shown in Figure 2d, the three siRNAs exhibited varied knockdown effects on HSP27 expression. In MARC-145 cells transfected with these siRNAs, viral N proteins were analyzed by Western blotting. As shown in Figure 2e, the knockdown of HSP27 by siRNA inhibited PRRSV-N expression compared with that of cells transfected with the siNC. As expected, the virus titer also decreased (Figure 2f). These results confirm that the inhibition of HSP27 synthesis leads to reduced PRRSV replication. Based on the above results, we concluded that HSP27 can promote PRRSV replication.

### 3.3. HSP27 Is Phosphorylated during PRRSV Infection

**Figure 3:**
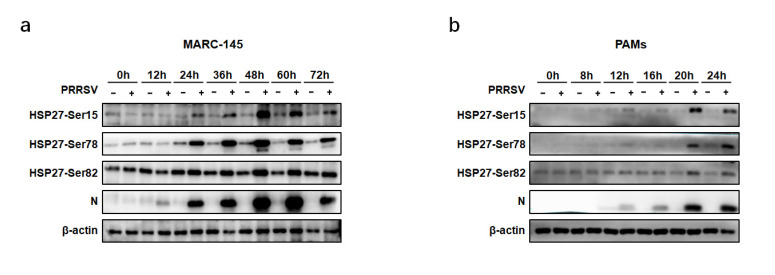
Phosphorylation of HSP27 during PRRSV infection. MARC-145 cells (**a**) or PAMs (**b**) were infected with PRRSV (MOI = 1.0). The cells were harvested at various time points as indicated. The expression levels of phosphorylated HSP27 were analyzed by Western blot.

Previous research showed that HSP27 could be phosphorylated at three serine (Ser) residues (Ser15, 78, and 82). Many stimuli, such as heat shock, injury and chemical stimulation can influence HSP27 phosphorylation. Phosphorylated forms of HSP27 can perform auxiliary roles [15]. Therefore, we explored the phosphorylation level of HSP27 during PRRSV infection. As shown in Figure 3, the levels of phosphorylation at Ser15, 78 and 82 of HSP27 were all upregulated during PRRSV replication in both MARC-145 cells and PAMs.

### 3.4. Suppressing the Phosphorylation of HSP27 Could Reduce PRRSV Replication and Viral dsRNA

**Figure 4:**
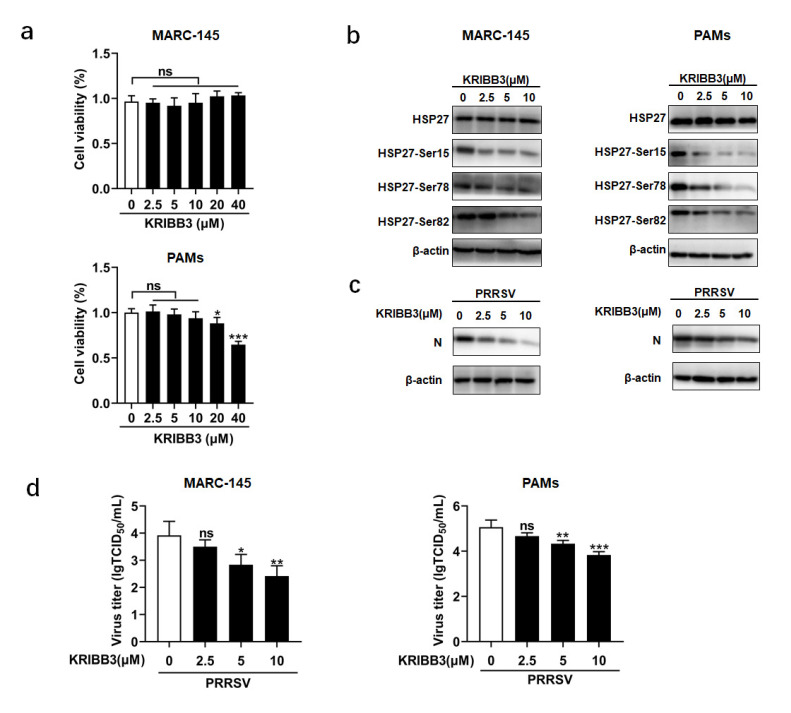
KRIBB3 inhibits PRRSV replication. (**a**) The cell toxicity of KRIBB3. MARC-145 cells or PAMs were incubated with different concentrations of KRIBB3 or DMSO for 48 h. Cell viability was evaluated using the CCK-8. (**b**–**d**) MARC-145 cells or PAMs were pretreated with various concentrations of KRIBB3 or DMSO for 24 h. The cells were then infected with PRRSV (MOI = 1.0). The infected cells were harvested at 24 hpi for Western blot analysis (**b**,**c**) and TCID_50_ assay (**d**). *p* < 0.05 (*), *p* < 0.01 (**), and *p* < 0.001 (***) were considered statistically significant.

Further investigations were performed to determine whether the phosphorylation state of HSP27 plays a role in PRRSV replication. KRIBB3 was used, which is a specific inhibitor that can inhibit HSP27 phosphorylation [19]. We first tested the cytotoxicity of KRIBB3 on MARC-145 cells and PAMs to determine the optimal concentration using a CCK-8. Cytotoxicity could not be detected in MARC-145 cells when 40 μM KRIBB3 was used. PAMs were more sensitive to KRIBB3, and toxicity could be found at a concentration of 20 μM (Figure 4a).

**Figure 5:**
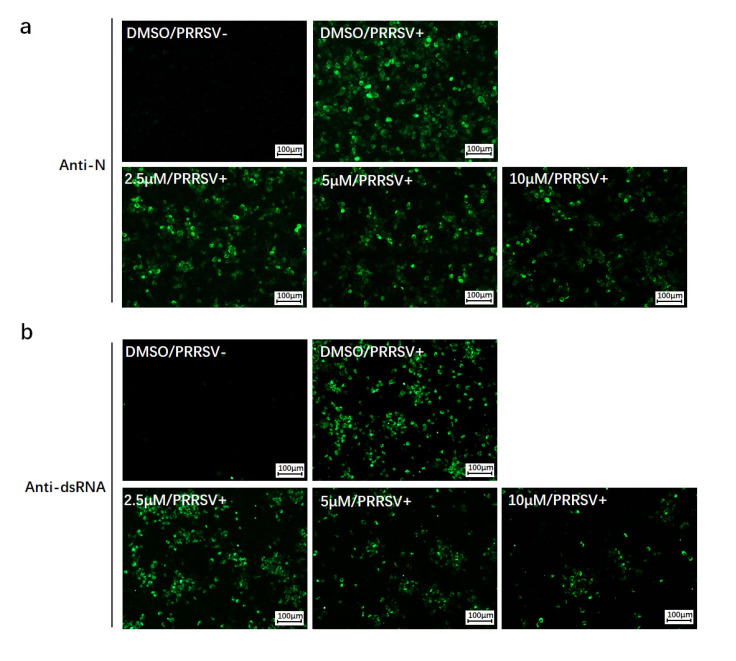
KRIBB3 blocked viral protein expression and reduced viral dsRNA. MARC-145 cells were pretreated with various concentrations of KRIBB3 or DMSO. After 24 h, the cells were infected or mock-infected with PRRSV (MOI = 1.0). The cells were fixed at 24 hpi. IFA was then performed with anti-N antibody (**a**) or anti-dsRNA (J2) antibody (**b**) and Alexa Fluor 488-conjugated (green) goat anti-mouse IgG. Bar, 100 μm.

MARC-145 cells or PAMs were then incubated with various concentrations of KRIBB3 for 24 h, followed by PRRSV infection for another 24 h. Considering the half-life of KRIBB3, the medium was refreshed every 24 h. As shown in Figure 4b, compared to the control groups, HSP27 phosphorylation was blocked in a dose-dependent manner in the groups treated with KRIBB3. Western blot analysis was performed to detect the level of viral N protein and viral titers were measured at 24 hpi (Figure 4c,d). In addition, IFA was also performed on MARC-145 cells (Figure 5a). We observed that KRIBB3 inhibited the expression of the N protein in a dose-dependent manner and reduced PRRSV progeny production. The results indicated that suppression of HSP27 phosphorylation reduced PRRSV replication.

During PRRSV replication, dsRNA can be generated and included in the RTCs. Therefore, dsRNA can be used as a marker to determine the formation of PRRSV RTCs. IFA was used to detect the viral dsRNA generated while KRIBB3 was treated with a specific antibody (J2). The results showed that the dsRNA level was decreased by KRIBB3 in a dose-dependent manner (Figure 5b). This suggests that suppressing HSP27 phosphorylation might block the formation of PRRSV RTCs and thus affect PRRSV replication.

### 3.5. HSP27 Can Interact with Multiple PRRSV-nsps

**Figure 6:**
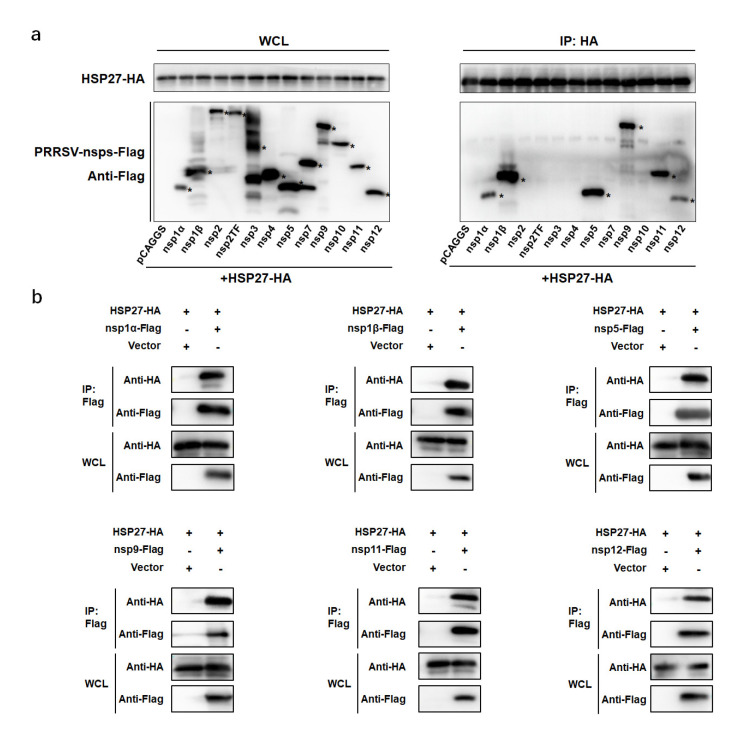
HSP27 interacts with multiple PRRSV-nsps. (**a**) HEK-293T cells were cotransfected with vectors encoding HA-tagged HSP27 and Flag-tagged PRRSV-nsps. After 48 h, the cells were lysed and immunoprecipitated with anti-HA antibodies. Whole-cell lysate (WCL) and immunoprecipitation (IP) complexes were used for immunoblotting with anti-HA and anti-Flag antibodies. Asterisks marked the expressed Flag-fusion PRRSV-nsps. (**b**) HEK-293T cells were cotransfected with Flag-tagged PRRSV-nsps and HA-tagged HSP27 expression vectors. At 48 h after transfection, cells were lysed and immunoprecipitated with an anti-Flag antibodies. WCL and IP complexes were used for immunoblotting analysis with anti-Flag and anti-HA antibodies.

A network of nsps is needed for the assembly of PRRSV RTCs during infection. To further explore the mechanism by which HSP27 mediates PRRSV replication, we screened the interactions between HSP27 and PRRSV-nsps through Co-IP assays. HEK-293T cells were cotransfected with pcDNA3.1-HSP27-HA and vectors encoding Flag-tagged PRRSV-nsps (except for nsp6 and nsp8). We found that HSP27 could interact with more than one nsps, including nsp1α, nsp1β, nsp5, nsp9, nsp11 and nsp12 (Figure 6a). In the reverse Co-IP experiment, all six nsps could efficiently coimmunoprecipitate with HSP27 (Figure 6b).

### 3.6. The Phosphorylation State of HSP27 Affects Its Interaction with PRRSV-nsps

**Figure 7:**
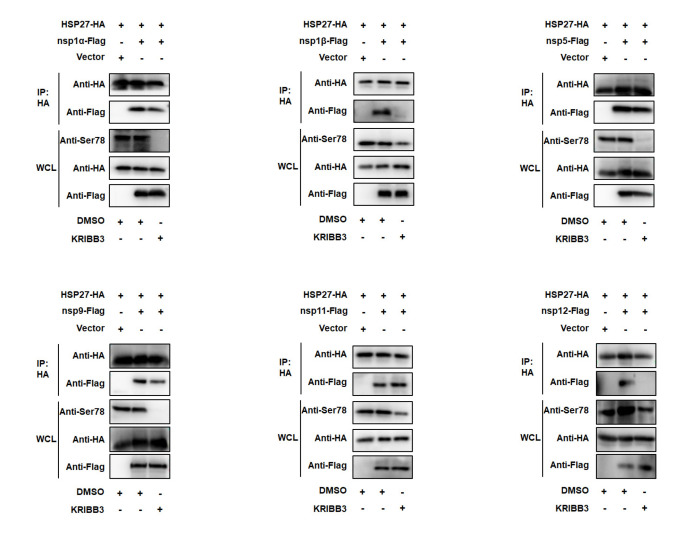
The phosphorylation state of HSP27 affects its interaction with PRRSV-nsps. HEK-293T cells were cotransfected with HA-tagged HSP27 and Flag-tagged PRRSV-nsps expression vectors. The cells were then incubated with DMSO or 10 μM KRIBB3 at 24 h after transfection. At 48 h after transfection, cells were lysed and immunoprecipitated with anti-HA antibodies. WCL and IP complexes were analyzed by immunoblotting with anti-HA and anti-Flag antibodies. The phosphorylation inhibition effect of KRIBB3 on HSP27 was tested with an anti-phospho-HSP27 (Ser78) antibody.

Considering that HSP27 can be phosphorylated during PRRSV infection and that suppressing the phosphorylation of HSP27 reduced virus replication, we then analyzed the effect of HSP27 phosphorylation on its interaction with viral nsps. We tested the cytotoxicity of KRIBB3 on HEK-293T cells. Toxicity could not be found below 10 μM (Figure S1). Therefore, future experiments were performed with KRIBB3 at 10 μM. HEK-293T cells were cotransfected with HSP27 and PRRSV-nsps expression vectors and then incubated with DMSO or 10 μM KRIBB3. As shown in Figure 7, KRIBB3 almost completely interrupted the interaction of HSP27 with nsp1β and nsp12 and could influence the interaction of HSP27 with nsp1α, nsp5, and nsp9 to some extent. However, the interaction between HSP27 and nsp11 was not influenced by KRIBB3. 

## 4. Discussion

PRRSV has plagued the world’s swine industry for more than 30 years [20,21]. It is a very successful virus, and no effective countermeasures exist. Therefore, researchers have attempted to target cellular proteins to uncover potential antiviral strategies. HSP27 (or HSPB1) is a member of the small HSPs family, which work as molecular chaperones and interact with a large number of proteins [22]. HSP27, as a multifunctional protein, participates in several cellular processes and has been demonstrated to be involved in various viral replications. HSP27 is expressed differently and plays different roles during different viral infections. For instance, HSP27 is induced in response to porcine circovirus type 2 (PCV2), enterovirus A71 (EV-A71) and herpes simplex virus type 1 (HSV-1) infection and can facilitate the replication of these viruses [16,23,24]. In contrast, HSP27 can be reduced by classical swine fever virus (CSFV), porcine epidemic diarrhea virus (PEDV) and hepatitis B virus (HBV), and functions as an antiviral effector during virus replication [17,25,26]. A previous proteomics analysis revealed that HSP27 was upregulated in PRRSV-infected PAMs [12]. Another study demonstrated that HSP27 is present in highly purified PRRSV virions [27]. It is reasonable to postulate that HSP27 may also be functionally involved in the PRRSV life cycle. In this study, we demonstrated that HSP27 was upregulated during PRRSV infection in both MARC-145 cells and PAMs. This regulation process was not PRRSV strain dependent. The UV-inactivated and heat-inactivated PRRSV showed no induction on HSP27 expression, which proved that the induction of HSP27 by PRRSV relied on virus replication. To further explore the role of HSP27 during PRRSV infection, HSP27 expression was modulated to analyze the effect on PRRSV replication. We observed that HSP27 overexpression resulted in an increase in PRRSV replication. On the other hand, the downregulation of HSP27 significantly reduced the level of PRRSV production. This means that HSP27 can facilitate the replication of PRRSV.

HSP27 contains an N-terminal WDPF domain, a highly conserved α-crystallin domain, and a C-terminal domain. A variety of stimuli can induce the phosphorylation of Ser 15, 78, and 82 in the N-terminal WDPF domain of HSP27. The auxiliary functions of HSP27 can be regulated by the oligomeric state, and its oligomerization is regulated by phosphorylation at the three residues [15]. Previous studies have suggested that increased HSP27 phosphorylation occurs during some viral infections. For example, increased phosphorylation at Ser15 and 78 of HSP27 was found during PCV2 infection [16]. Respiratory syncytial virus (RSV) infection was concomitant with increased HSP27 phosphorylation on both Ser78 and 82 (Ser15 was not examined) [28]. HSV-1 infection causes rapid HSP27 phosphorylation in a p38-dependent fashion [29]. Epstein–Barr virus (EBV) infection upregulates HSP27 phosphorylation via the PI3K/Akt pathway [30]. In our study, we found that the phosphorylation levels at Ser15, 78 and 82 of HSP27 were all upregulated during PRRSV replication. Considering that the total HSP27 levels were also upregulated, it is difficult to determine whether the upregulated phosphorylation was due to the change in the total HSP27 expression level. However, it can be postulated that the phosphorylation levels at Ser15 and 78 were definitely upregulated at 24 hpi in MARC-145 cells, since the total HSP27 level remained unchanged at this time point (Figure 1b). 

To further clarify the role of HSP27 phosphorylation during PRRSV replication, KRIBB3, a specific chemical inhibitor, was used to inhibit HSP27 phosphorylation. The results showed that suppressing HSP27 phosphorylation reduced PRRSV replication. As a positive-strand RNA virus, the synthesis of viral RNA is directed by RTCs, which are mainly composed of a network of PRRSV-nsps and some cellular proteins. During viral replication, dsRNA can be generated as a replicative intermediate contained in the viral RTCs and can be used as a marker to examine the formation of RTCs. In our study, the formulation of viral dsRNA was found to be reduced in KRIBB3-treated cells. This result suggested that HSP27 might be involved in the network of PRRSV-nsps to form RTCs and thus affect viral replication. Co-IP was then performed to address the interactions of HSP27 with PRRSV-nsps. Here, we found that HSP27 could interact with six PRRSV-nsps, including nsp1α, nsp1β, nsp5, nsp9, nsp11, and nsp12. Among these nsps, nsp5 is a transmembrane protein, together with nsp2 and nsp3, that recruits other components of PRRSV RTCs to the replicase site. Nsp9, nsp11, and nsp12 are thought to be the core components of RTCs. A complex interaction network of nsps is necessary to assemble PRRSV RTCs. Nsp12 was demonstrated to be a novel interaction hub that connects these nsps, including nsp1α and nsp1β. We then postulated that HSP27, as a molecular chaperone, might assist these nsps in translocating into the RTCs.

Our study also revealed that suppressing HSP27 phosphorylation with KRIBB3 could interrupt the interaction of HSP27 with these nsps (except for nsp11). Notably, the interactions of HSP27 with nsp1β and nsp12 were completely disrupted by KRIBB3. This result may explain why the suppression of HSP27 phosphorylation could reduce the formulation of RTCs and virus replication. During the experiment, we also found that nsp12 could induce the phosphorylation of HSP27 (Figure 7). Regarding its function, Nsp12 is among the least known nsps of PRRSV. This finding enriched the function of nsp12 and revealed that it could modulate HSP27, a cellular factor. Further studies are needed to clarify the kinases and phosphatases that regulate the phosphorylation pattern of HSP27 during this process.

## 5. Conclusions

In summary, our study demonstrated that PRRSV replication can induce HSP27 expression and phosphorylation *in vitro*. HSP27 can interact with multiple PRRSV-nsps and promote viral replication. Suppressing the phosphorylation of HSP27 can interrupt its interaction with PRRSV-nsps and might disrupt the formation of RTCs. These results reveal that HSP27 plays an important role in PRRSV replication.

## Data Availability

The data presented in this study are available in the article.

## Supplementary Materials

The following supporting information can be downloaded at: https://www.mdpi.com/article/10.3390/pathogens12010091/s1, Figure S1: The cell toxicity of KRIBB3 on HEK-293T cells.

## References

[1] Rahe M.C., Murtaugh M.P. (2017). Effector mechanisms of humoral immunity to porcine reproductive and respiratory syndrome virus. Vet. Immunol. Immunopathol..

[2] Fang Y., Treffers E.E., Li Y., Tas A., Sun Z., van der Meer Y., de Ru A.H., van Veelen P.A., Atkins J.F., Snijder E.J. (2012). Efficient −2 frameshifting by mammalian ribosomes to synthesize an additional arterivirus protein. Proc. Natl. Acad. Sci. USA.

[3] Kappes M.A., Faaberg K.S. (2015). PRRSV structure, replication and recombination: Origin of phenotype and genotype diversity. Virology.

[4] Fang Y., Snijder E.J. (2010). The PRRSV replicase: Exploring the multifunctionality of an intriguing set of nonstructural proteins. Virus Res..

[5] Nan H., Lan J., Tian M., Dong S., Tian J., Liu L., Xu X., Chen H. (2018). The Network of Interactions Among Porcine Reproductive and Respiratory Syndrome Virus Non-structural Proteins. Front. Microbiol..

[6] Giedroc D.P., Cornish P.V. (2009). Frameshifting RNA pseudoknots: Structure and mechanism. Virus Res..

[7] Li Y., Treffers E.E., Napthine S., Tas A., Zhu L., Sun Z., Bell S., Mark B.L., van Veelen P.A., van Hemert M.J. (2014). Transactivation of programmed ribosomal frameshifting by a viral protein. Proc. Natl. Acad. Sci. USA.

[8] Johnson C.R., Griggs T.F., Gnanandarajah J., Murtaugh M.P. (2011). Novel structural protein in porcine reproductive and respiratory syndrome virus encoded by an alternative ORF5 present in all arteriviruses. J. Gen. Virol..

[9] Yuan S., Murtaugh M.P., Schumann F.A., Mickelson D., Faaberg K.S. (2004). Characterization of heteroclite subgenomic RNAs associated with PRRSV infection. Virus Res..

[10] Song J., Liu Y., Gao P., Hu Y., Chai Y., Zhou S., Kong C., Zhou L., Ge X., Guo X. (2018). Mapping the Nonstructural Protein Interaction Network of Porcine Reproductive and Respiratory Syndrome Virus. J. Virol..

[11] Gao J., Xiao S., Liu X., Wang L., Ji Q., Mo D., Chen Y. (2014). Inhibition of HSP70 reduces porcine reproductive and respiratory syndrome virus replication in vitro. BMC Microbiol..

[12] Zhang H., Guo X., Ge X., Chen Y., Sun Q., Yang H. (2009). Changes in the cellular proteins of pulmonary alveolar macrophage infected with porcine reproductive and respiratory syndrome virus by proteomics analysis. J. Proteome Res..

[13] Gao J., Xiao S., Liu X., Wang L., Zhang X., Ji Q., Wang Y., Mo D., Chen Y. (2014). Inhibition of HSP90 attenuates porcine reproductive and respiratory syndrome virus production in vitro. Virol. J..

[14] Jacob P., Hirt H., Bendahmane A. (2017). The heat-shock protein/chaperone network and multiple stress resistance. Plant Biotechnol. J..

[15] Singh M.K., Sharma B., Tiwari P.K. (2017). The small heat shock protein Hsp27: Present understanding and future prospects. J. Biol..

[16] Liu J., Zhang L., Zhu X., Bai J., Wang L., Wang X., Jiang P. (2014). Heat shock protein 27 is involved in PCV2 infection in PK-15 cells. Virus Res..

[17] Sun M., Yu Z., Ma J., Pan Z., Lu C., Yao H. (2017). Down-regulating heat shock protein 27 is involved in porcine epidemic diarrhea virus escaping from host antiviral mechanism. Vet. Microbiol..

[18] Ke W., Fang L., Jing H., Tao R., Wang T., Li Y., Long S., Wang D., Xiao S. (2017). Cholesterol 25-Hydroxylase Inhibits Porcine Reproductive and Respiratory Syndrome Virus Replication through Enzyme Activity-Dependent and -Independent Mechanisms. J. Virol..

[19] Li J., Qi X., Jiang B., Huang T., Luo L., Liu S., Yin Z. (2019). Phosphorylated Heat Shock Protein 27 Inhibits Lipopolysaccharide-Induced Inflammation in Thp1 Cells by Promoting TLR4 Endocytosis, Ubiquitination, and Degradation. Inflammation.

[20] Albina E. (1997). Porcine reproductive and respiratory syndrome: Ten years of experience (1986–1996) with this undesirable viral infection. Vet. Res..

[21] Neumann E.J., Kliebenstein J.B., Johnson C.D., Mabry J.W., Bush E.J., Seitzinger A.H., Green A.L., Zimmerman J.J. (2005). Assessment of the economic impact of porcine reproductive and respiratory syndrome on swine production in the United States. J. Am. Vet. Med. Assoc..

[22] Gusev N.B., Bogatcheva N.V., Marston S.B. (2002). Structure and properties of small heat shock proteins (sHsp) and their interaction with cytoskeleton proteins. Biochemistry.

[23] Mathew S.S., Della S.M., Burch A.D. (2009). Modification and reorganization of the cytoprotective cellular chaperone Hsp27 during herpes simplex virus type 1 infection. J. Virol..

[24] Dan X., Wan Q., Yi L., Lu J., Jiao Y., Li H., Song D., Chen Y., Xu H., He M.L. (2019). Hsp27 Responds to and Facilitates Enterovirus A71 Replication by Enhancing Viral Internal Ribosome Entry Site-Mediated Translation. J. Virol..

[25] Ling S., Luo M., Jiang S., Liu J., Ding C., Zhang Q., Guo H., Gong W., Tu C., Sun J. (2018). Cellular Hsp27 interacts with classical swine fever virus NS5A protein and negatively regulates viral replication by the NF-kappaB signaling pathway. Virology.

[26] Tong S.W., Yang Y.X., Hu H.D., An X., Ye F., Ren H., Li S.L., Zhang D.Z. (2013). HSPB1 is an intracellular antiviral factor against hepatitis B virus. J. Cell Biochem..

[27] Zhang C., Xue C., Li Y., Kong Q., Ren X., Li X., Shu D., Bi Y., Cao Y. (2010). Profiling of cellular proteins in porcine reproductive and respiratory syndrome virus virions by proteomics analysis. Virol. J..

[28] Kostenko S., Moens U. (2009). Heat shock protein 27 phosphorylation: Kinases, phosphatases, functions and pathology. Cell. Mol. Life Sci..

[29] Karaca G., Hargett D., McLean T.I., Aguilar J.S., Ghazal P., Wagner E.K., Bachenheimer S.L. (2004). Inhibition of the stress-activated kinase, p38, does not affect the virus transcriptional program of herpes simplex virus type 1. Virology.

[30] Fukagawa Y., Nishikawa J., Kuramitsu Y., Iwakiri D., Takada K., Imai S., Satake M., Okamoto T., Fujimoto M., Okita K. (2008). Epstein-Barr virus upregulates phosphorylated heat shock protein 27 kDa in carcinoma cells using the phosphoinositide 3-kinase/Akt pathway. Electrophoresis.

