# Supplementary figures and images for: HSP27 Interacts with Nonstructural Proteins of Porcine Reproductive and Respiratory Syndrome Virus and Promotes Viral Replication
Source: Pathogens. 2023 Jan 5;12(1):91. doi: 10.3390/pathogens12010091 (PMC9860683; doi:10.3390/pathogens12010091)

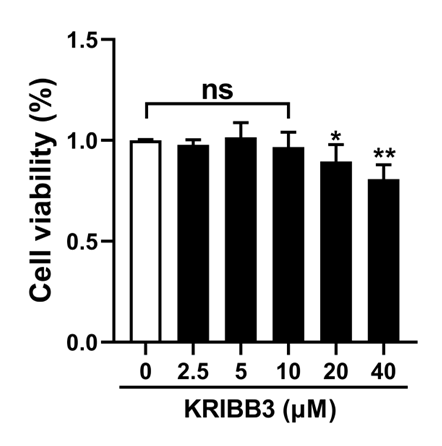

Supplement: Supplementary file 1 [file pathogens-12-00091-s001.zip › pathogens-2096538-supplementary.tif]
